# Supplementary figures and images for: Early Postnatal Neuroinflammation Produces Key Features of Diffuse Brain White Matter Injury in Rats
Source: Brain Sci. 2024 Sep 27;14(10):976. doi: 10.3390/brainsci14100976 (PMC11505921; doi:10.3390/brainsci14100976)

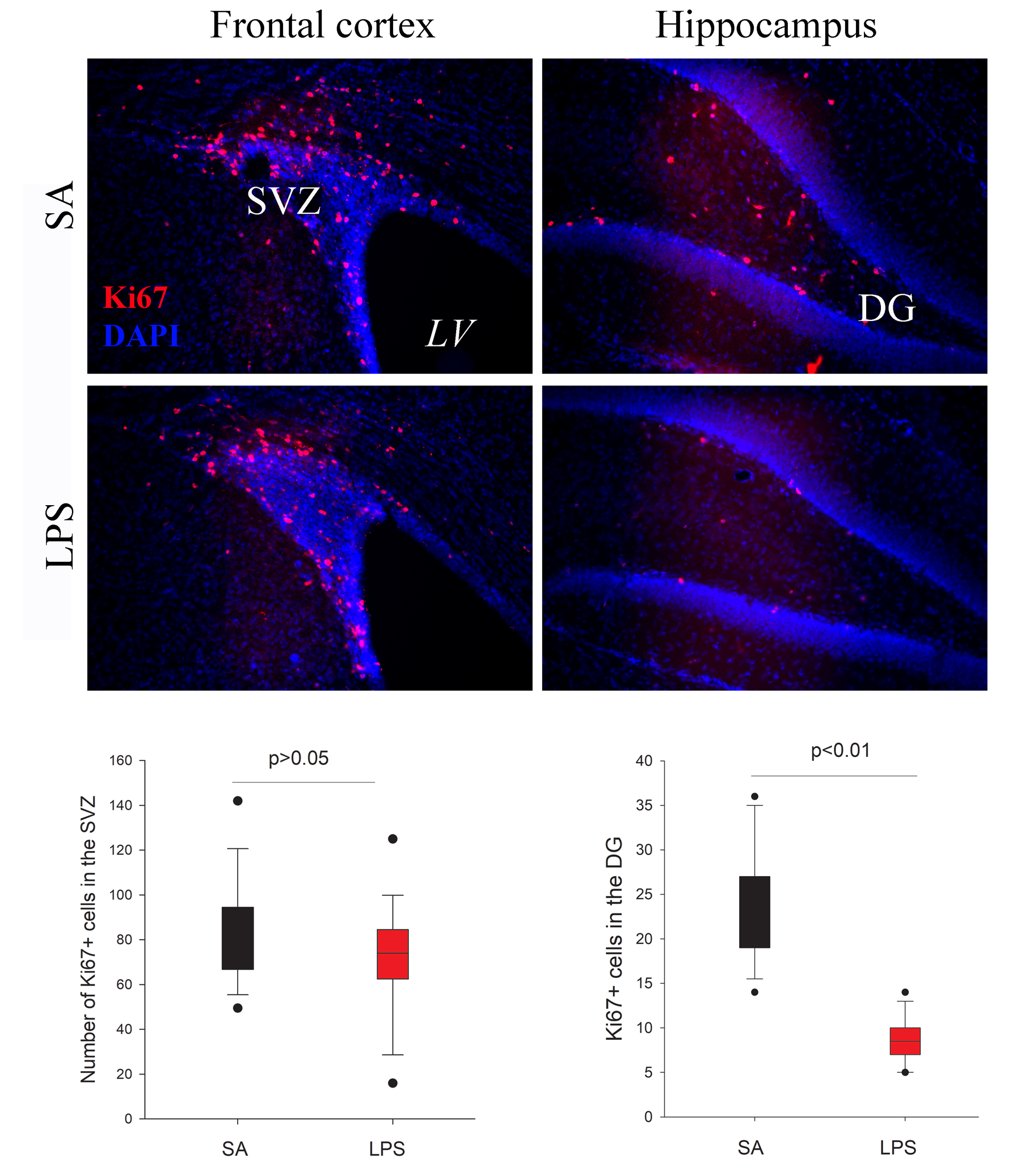

Supplement: Supplementary file 1 [file brainsci-14-00976-s001.zip › brainsci-3174513-supplementary.tif]
